# Sleep disturbance has the largest impact on children's behavior and emotions

**DOI:** 10.3389/fped.2022.1034057

**Published:** 2022-11-28

**Authors:** Michiko Matsuoka, Toyojiro Matsuishi, Shinichiro Nagamitsu, Mizue Iwasaki, Akiko Iemura, Hitoshi Obara, Yushiro Yamashita, Masaharu Maeda, Tatsuyuki Kakuma, Naohisa Uchimura

**Affiliations:** ^1^ Department of Pediatrics and Child Health, School of Medicine, Kurume University, Kurume, Japan; ^2^ Department of Neuropsychiatry, School of Medicine, Kurume University, Kurume, Japan; ^3^ Research Center for Children and Research Center for Rett Syndrome, St. Mary’s Hospital, Kurume, Japan; ^4^ Department of Pediatrics, Faculty of Medicine, Fukuoka University, Fukuoka, Japan; ^5^ Biostatistics Center, School of Medicine, Kurume University, Kurume, Japan; ^6^ Department of Disaster Psychiatry, Fukushima Medical University, Fukushima, Japan

**Keywords:** children, strength and difficulties questionnaire (SDQ), children’s sleep habits questionnaire (CSHQ), after school lessons, single mother families, path analysis

## Abstract

**Objective:**

Children's behavior and emotions are affected by sleep disturbances, the parent-child relationship, media viewing time, and the social status of parents and caregivers. We conducted a questionnaire survey to identify the factors that have the greatest impact on children's behavior and emotions and how these factors relate to each other.

**Methods:**

A parental questionnaire survey was performed at a public elementary school. The questionnaire comprised questions on the family environment (e.g., family structure, media and game exposure, after-school lessons, and caregiver's work schedule) and physical information, the Strengths and Difficulties Questionnaire (SDQ), the Children's Sleep Habits Questionnaire (CSHQ), and the Pittsburgh Sleep Quality Index (PSQI) for parents' sleep condition. A path diagram was drawn to hypothesize the complex interrelationships among factors, and structural equation modeling was used to estimate the path coefficients.

**Result:**

We identified several factors that significantly affected the SDQ score. The CSHQ total score had the largest impact, followed by after-school lessons, single-mother families, and children's sex. In addition, several indirect pathways that led to the CSHQ score (i.e., a pathway from time spent watching television to CSHQ score *via* children's bedtime and a pathway from single-mother family to CSHQ score *via* PSQI total score) significantly affected the SDQ score.

**Conclusion:**

Children's sleep habits that were influenced by several environmental factors had the greatest impact on children's behavior and emotions, which suggested that children's behavioral problems can be improved by interventions focused on sleep habits, such as sleep hygiene instructions.

## Introduction

Children's mental health, which includes daily activities, behaviors, emotions, and problems with peers, is an important element in their development of resilience and prosocial skills (1, 2). Children who visit the hospital because of mental health problems, which include behavioral or emotional problems, often have multiple complex underlying factors, such as physical disorder, problems with parent-child lifestyles, sleep disturbances, addiction to media and games, and economic status. Studies have shown that each of these underlying factors affects children's behavior and emotions (3–11). Niclasen et al. reported that children with pediatric hearing loss had significantly more behavioral problems than those without, such as hyperactivity and difficulties with reading and writing. Moreover, Smedje et al. reported that sleep disturbance was associated with emotional and behavioral problems (3, 4). Furthermore, tossing and turning during sleep and sleepwalking are significantly correlated with hyperactivity, and night terrors and difficulty falling asleep are significantly correlated with children's emotional symptoms (4). To determine how to treat behavioral and emotional problems in children with multiple underlying problems, it is crucial to identify the factors that have the greatest impact on the behavior and emotions of children.

The Strength and Difficulties Questionnaire (SDQ) is a short screening instrument that addresses not only children's difficulties but also the social orientation of infants, children, and adolescents (12). The SDQ is widely used in medical and educational fields and has shown to be associated with physical and sleep disorders in children (3–8, 10). Furthermore, our previous study revealed that the SDQ is a useful screening tool to determine the behavioral and emotional characteristics of attention deficit hyperactivity disorder (ADHD) and autism spectrum disorder (ASD) (13). Although several reports have investigated the condition of children in clinics and hospitals to understand the effects of sleep status on children's emotions/behavior and promote children's growth and development, the analysis of children who visit hospitals and clinics is biased and does not provide a general real-world understanding (14–17). Therefore, our study included all students in an elementary school, assessed children's behavioral and emotional problems using the SDQ, and investigated the relationship between physical factors, children's lifestyles, and the caregiver environment.

The purpose of this study was to identify the factors affecting children that have the greatest impact on children's behavior and emotional problems and to determine how each factor influences another. We explored various factors that influence children's emotions and behaviors, such as children's physical condition, children's sleep status, time spent watching television, playing games, using the internet, family structure, family employment status, and family sleep status. For clinical relevance, we developed a structural model of how these factors influence each other, which was verified using path analysis.

## Materials and methods

### Subjects

A parental questionnaire was distributed between September 2010 and October 2010 at one public elementary school in Kurume, Japan, to 436 students from Grades 1 to 6, ranging in age from 6 to 12 years. Forty-eight children with developmental disorders, which included ASD, ADHD, and learning disorders, were excluded from the study because of their high needs for managing emotional and behavioral problems (13). Therefore, a total of 388 students were included as candidates for this study.

### Survey procedure

The design of the study and the procedures for obtaining informed consent were approved by the Medical Ethics Committee of School of Medicine, Kurume University (No. 10110). Informed consent was obtained from each child's parents prior to their participation in the study. A set of questionnaires, which included an instruction letter and an individual information sheet to provide physical information, children's extracurricular activities, family structure, working condition of the parents or caregivers, the Japanese version of the SDQ (18, 19), the Japanese version of the Children's Sleep Habits Questionnaire (CSHQ) (20, 21), and the Japanese version of the Pittsburgh Sleep Quality Index (PSQI) (22, 23), was handed to all students. Students' parents or caregivers were required to complete the questionnaires in accordance with the instructions provided.

### Participants' physical information and family environment

Information regarding physical and family environmental factors was obtained using the set of questionnaires filled out by the children's parents or caregivers. Physical factors included children's age, children's sex, physical illness (e.g., allergic disorder, adenoids, and epilepsy), and daily medication use. Lifestyle was evaluated using questions about children's extracurricular activities (e.g., regular participation in sports, such as swimming, ballet, and football, and cultural activities, such as learning Japanese calligraphy, piano, and English) and media exposure (e.g., television viewing habits, playing video games, and/or using the internet during both weekdays and weekends). Parents and caregivers were also asked to provide information on their parenting environment, such as family structure, work, the time they return home from work, and irregular shift work.

### Measurements

#### The strength and difficulty questionnaire

The SDQ is a short screening instrument that evaluates the positive and negative behavioral attributes of infants, children, and adolescents (12). The SDQ includes 25 items, each of which is rated as being not true (0), somewhat true (1), or certainly true (2). Each SDQ subscale consists of five items, yielding a total score between 0 and 10. Although the wording chosen for 10 of the 25 SDQ questions addresses positive behavioral attributes, five of these 10 item scores are inverted before being totaled. Thus, four of the SDQ subscales represent problem scores (i.e., emotional symptoms, conduct problems, hyperactivity/inattention, and peer problems), which are summed to provide a total difficulties score ranging from 0 to 40. The fifth subscale assesses the positive aspect of prosocial behavior. Higher scores on the four subscales reflect greater difficulties, whereas higher scores on the prosocial behavior subscale reflect higher prosociality. The SDQ has been translated into numerous languages and is widely used across several countries (18, 19, 24–27). The Japanese version of SDQ was translated and standardized in 2008 (18) in a community sample of 2,899 people. In this study, we used the recommended banding and divided participants into three groups: normal, borderline, and clinical range. Additionally, we confirmed that the SDQ distribution was not significantly different from that of the community sample used by Matsuishi et al. (18).

#### The children's sleep habits questionnaire

The CSHQ is a parent-reported instrument used to assess sleep patterns and sleep problems in children aged 4–10 years (20). It contains 56 items related to common sleep behaviors in children. A sleep/wake pattern is established for each child based on information provided by the parents or caregivers based on the time the child goes to sleep (sleep start time), the time the child wakes up (sleep end time), and the duration of sleep. Furthermore, parents or caregivers are asked to recall the child's sleep behaviors during a recent week. The items on the CSHQ are rated on a three-point Likert scale as follows: “usually” if the sleep behavior occurred five to seven times/week; “sometimes” if the behavior occurred two to four times/week; and “rarely” if the behavior did not occur or occurred only once a week. The total score (48 items) and the eight subscale scores (33 items) reflect sleep domains that encompass sleep disturbances experienced by this age group. The subscales provide measures of different aspects of sleep, such as bedtime resistance, sleep onset delay, sleep duration, sleep anxiety, night-time waking, parasomnias, sleep-disordered breathing, and daytime sleepiness. Higher scores indicate more sleep problems. A total CSHQ score of 41 has been reported to be a sensitive clinical cutoff score for the identification of probable sleep problems. The CSHQ has been translated, standardized, and widely used in various countries (21, 28–30).

#### The pittsburgh sleep quality index

The PSQI is a self-rated questionnaire that assesses sleep quality and disturbances over a 1-month period (22). In the present study, the PSQI was used to assess the sleep quality of children's parents or caregivers. Nineteen items of the PSQI are used to generate seven component scores: subjective sleep quality, sleep latency, sleep duration, habitual sleep efficiency, sleep disturbances, use of sleeping medication, and daytime dysfunction. The summed score of these seven components yields a total score. The Japanese version of the PSQI was validated in 2000 (21). Using a cutoff point of 5.5 for the total PSQI score achieved 85.7% sensitivity and 86.6% specificity for primary insomnia.

### Statistical analysis

T-tests were used to examine the factors (i.e., after-school activities, time spent watching television, playing video games, and using the internet, family structure, and income source) related to the child-rearing environment by sex.

The representativeness of participants (*n* = 365) was assessed by comparing the distribution of the SDQ score of participants with that of a community sample (*n* = 2,899), which was used as a reference population. To this end, equivalence between the two multinomial distributions was tested using the goodness-of-fit *χ*^2^-test (31), with a significance level of 5% and an equivalence margin of 0.0225 (defined as the sum of squared distance between the two proportions).

The primary aim of the data analyses was to identify factors that affect the SDQ score. Initially, many factors were considered interrelated; therefore, we decided that the statistical methods for evaluating the effects of the factors on SDQ score should reflect clinically relevant perspectives. To this end, a subset of factors was divided into three groups: “Physical factors,” “Lifestyle,” and “Parenting environment.” CSHQ total score, PSQI total score, children's bedtime, and parents’ bedtime were treated as a single factor. Children's and parents' bedtimes were defined as deviations (in minutes) from a reference time, which were 10 PM and 11 PM for children and parents, respectively.

**Figure 1:**
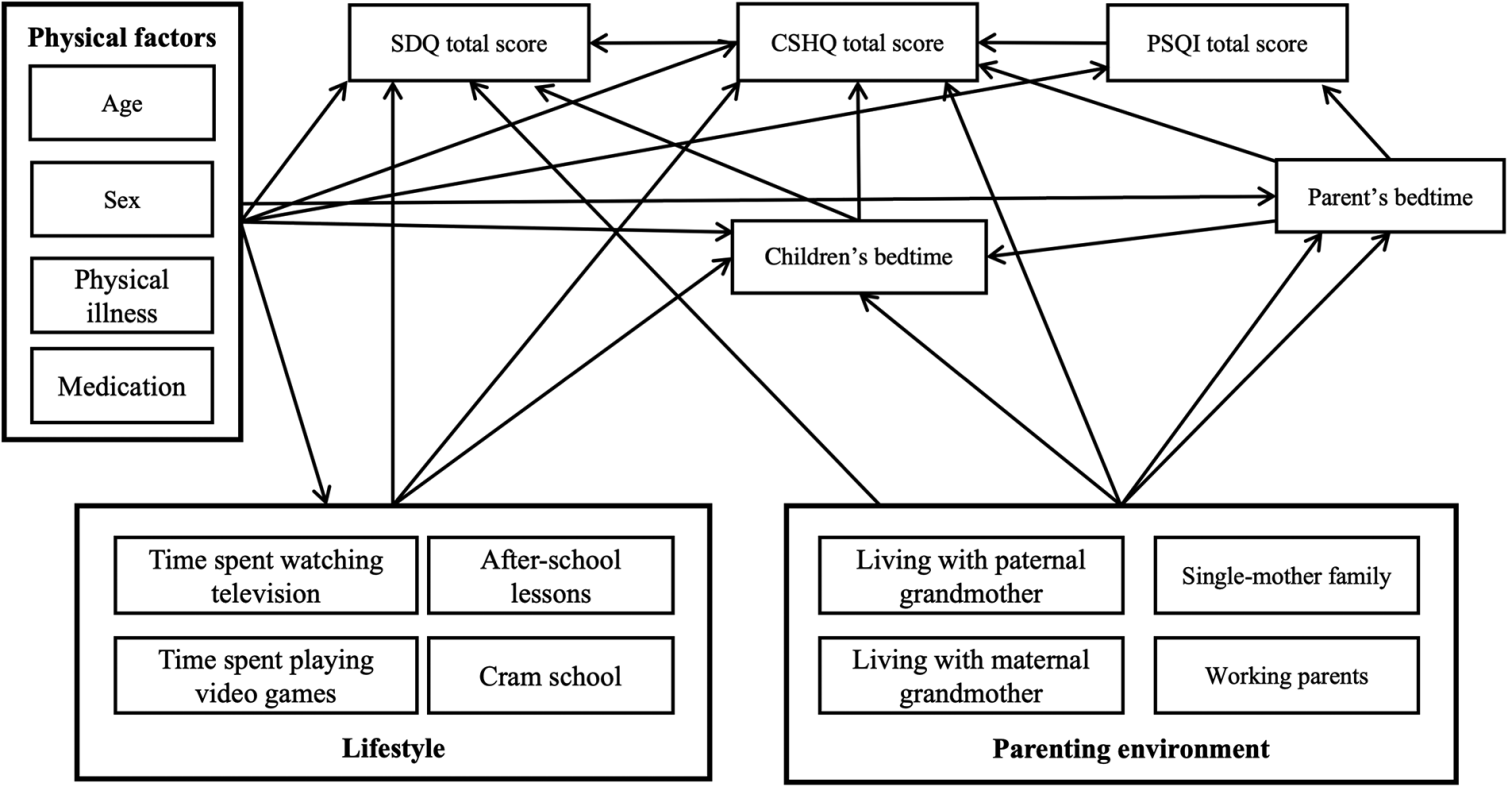
Path diagram hypothesizing the complex interrelationships among factors. We divided non-sleep-related factors that may affect the SDQ total score into physical, lifestyle, and parenting environment factors. SDQ, Strengths and Difficulties Questionnaire; CSHQ, Children’s Sleep Habits Questionnaire; PSQI, Pittsburgh Sleep Quality Index.

**Figure 2:**
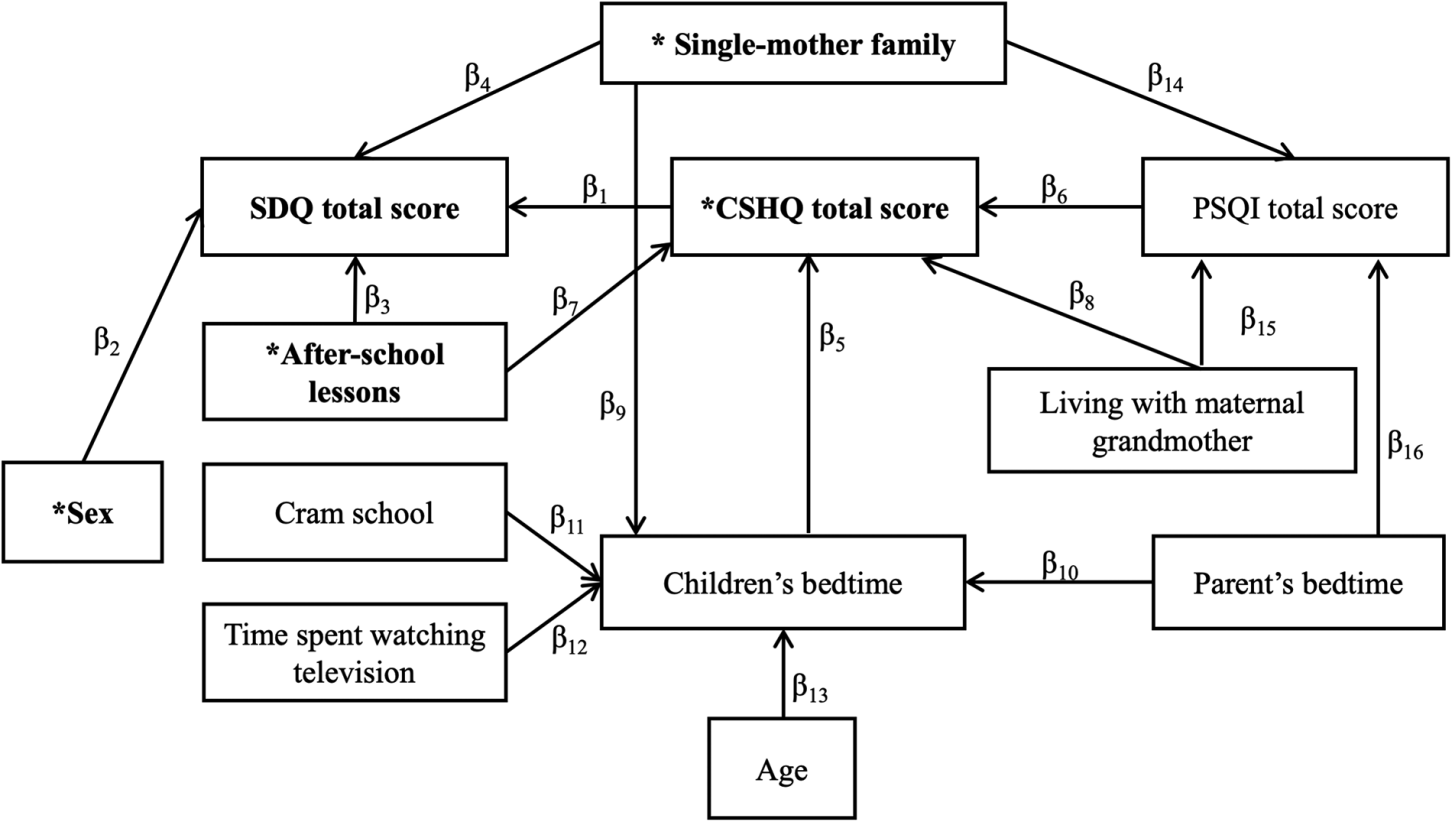
Path diagram. Path diagram of factors that affect the SDQ total score. *The four factors that had a direct effect on the SDQ total score. SDQ, Strengths and Difficulties Questionnaire; CSHQ, Children's Sleep Habits Questionnaire; PSQI, Pittsburgh Sleep Quality Index.

As depicted in Figure 1, a path diagram was drawn to hypothesize the complex interrelationship among factors. Structural equation modeling was used to estimate path coefficients. Because the factors within each group are expected to be highly correlated, variable selection was carried out in an exploratory manner. Specifically, various structural equation models, such as those with all factors included or those including only a single factor from each of the three groups, were fitted, and factors with a *p*-value for the coefficient of >0.5 were eliminated. After several iterations of model fitting, a final path diagram was constructed as shown in Figure 2. The effects of the factors were expressed as direct and total effects. The magnitude of each factor's effect on the SDQ total score was compared using standardized parameter estimates. Furthermore, the effect of the four factors of the SDQ subscale was estimated using a multivariate regression model. All statistical analyses were performed using STATA/MP 16.1 (StataCorp LLC, College Station, Texas).

## Results

### Participant characteristics

Participant characteristics are shown in Table 1. A total of 370 students (age range 6–12 years; 182 boys and 188 girls) participated in the study, which yielded a response rate of 95.4% (370/388). The number (percentage) of children who had a physical disorder was 74 (20.0%), which included 18 with asthma (4.9%), 35 with an allergy (9.5%), 5 with adenoids (1.4%), 2 with epilepsy (0.5%), and 2 with type 1 diabetes (0.5%). There were 29 (7.8%) children who were on medication: 22 were on antiallergic medication (5.9%), two were on antipsychotic medication (0.5%), two were on antiepileptic medication (0.5%), and two were on antidiabetic medication (0.5%).

**Table 1:** Participant characteristics.

	*n* = 370
Age (years)	9.4 ± 4.5
Sex	
Boys: *N* (%)	182 (49.2%)
Girls: *N* (%)	188 (50.8%)
BMI (kg/m^2^)	16.8 ± 2.9
Physical disorders	77 (20.8%)
Asthma	18 (4.9%)
Allergy	35 (9.5%)
Adenoids	5 (1.4%)
Epilepsy	2 (0.5%)
Type 1 diabetes	2 (0.5%)
Medications	29 (7.8%)
Antiallergic medicines	22 (5.9%)
Antipsychotic medicines	2 (0.3%)
Antiepileptic medicines	2 (0.5%)
Antidiabetic medicines	2 (0.5%)

Data are presented as means ± SDs or numbers of children with percentages in parentheses, as appropriate.

BMI, body mass index; SD, standard deviation.

### Participants' extracurricular activities, family structure, and family employment status

The number (percentage) of children attending cram school was 106 (28.6%), and the number of children and frequency of attendance showed a trend increase in those in a higher grade (data not shown). No significant difference was found between boys and girls for cram school attendance. The number of children who attended after-school lessons was 276 (74.8%), and there was no sex difference. The number of children attending cultural lessons, such as piano, Japanese calligraphy, and English conversational lessons, was 192 (51.9%), and significantly more girls attended these lessons than did boys. For sports lessons, such as swimming, baseball, and soccer, 197 (53.4%) children participated, and significantly more boys took part in these activities than did girls. Girls spent longer playing video games than boys on Saturdays. In addition, boys spent more time playing video games than girls on weekdays and weekends. There was no significant difference between boys and girls in terms of time spent using the internet. In terms of family structure and income source, 51 (13.8%) were single-parent families, and 69 (18.6%) had no siblings. There were 138 (36.9%) dual-income families, and the number of children whose parents worked irregular shifts was 70 (18.9%). No significant difference was observed between boys and girls in family structure or income source. Results are summarized in Table 2.

**Table 2:** Participants’ extracurricular activities, family structure, and family employment status.

	*n* = 370	Boys (*n* = 182)	Girls (*n* = 185)
After-school activities			
Cram school	106 (28.6%)	55	51
After-school lessons	276 (74.8%)	135	141
Culture lessons	192 (51.9%)	76	116**
Sport lessons	197 (53.4%)	117	80**
Time spent watching television (h)			
Weekdays	2.1 ± 1.2	2.0 ± 1.1	2.1 ± 1.4
Saturdays	3.1 ± 1.8	2.8 ± 1.7	3.3 ± 1.9*
Sundays	3.2 ± 1.9	3.1 ± 1.8	3.4 ± 2.0
Time spent playing video games (h)			
Weekdays	0.5 ± 0.7	0.7 ± 0.8	0.3 ± 0.5**
Saturdays	1.1 ± 1.9	1.3 ± 1.2	0.9 ± 2.3**
Sundays	1.1 ± 1.9	1.4 ± 1.4	0.9 ± 2.3**
Time spent using the internet (h)			
Weekdays	0.1 ± 0.3	0.06 ± 0.3	0.05 ± 0.3
Saturdays	0.1 ± 0.4	0.08 ± 0.3	0.12 ± 0.5
Sundays	0.1 ± 0.4	0.10 ± 0.4	0.12 ± 0.5
Family structure			
Single-parent family	51 (13.8%)	25	26
Single-mother family	44 (11.9%)	22	22
Single-father family	7 (1.9%)	3	4
Child without sibling	69 (18.6%)	34	35
Child with older brother	104 (28.2%)	50	54
Child with older sister	99 (26.8%)	49	50
Child with younger brother	89 (24.1%)	41	48
Child with a younger sister	94 (25.5%)	46	48
Living with grandparents	84 (22.7%)	42	42
Income source			
Dual income	138 (36.9%)	67	71
Shift worker	70 (18.9)	31	39

Data are presented as means ± SDs or as numbers of children with percentages in parentheses, as appropriate. *p < 0.05, **p < 0.01 boys vs. girls.

### Distributions of the SDQ score in the study participants and the community sample

Table 3 shows the SDQ scores of the study participants and the community sample. Of the study participants, 43 (11.8%) had a clinical score (≥16) for SDQ total difficulties. The percentages of children who scored above the cutoff scores of the SDQ subscales were 8.5% for emotional symptoms, 9.3% for conduct problems, 9.3% for hyperactivity, 9.0% for peer problems, and 13.2% for prosocial behavior. The comparison of the distribution of SDQ score between the study participants and that of the community sample showed that the distributions of all six subscales of SDQ were equivalent (Table 3).

**Table 3:** Distribution of SDQ score in the study participants and the community sample.

	Participant, n (%) *n* = 365			Community sample (%)^a^ *n* = 2899		
	Normal	Border	Clinical	Normal	Border	Clinical
Total difficulty	280 (76.7)	42 (11.5)	43 (11.8)	80.6	9.9	9.5
Emotional symptoms	304 (83.1)	30 (8.2)	31 (8.5)	84.3	7.2	8.5
Conduct problems	297 (81.4)	34 (9.3)	34 (9.3)	84.3	8.6	7.1
Hyperactivity	299 (81.9)	32 (8.8)	34 (9.3)	83.6	6.8	9.7
Peer problems	300 (82.2)	32 (8.8)	33 (9.0)	90.1	5.5	4.4
Prosocial behavior	257 (70.4)	60 (16.4)	48 (13.2)	71.2	15.5	13.3

^a^ (18).

### Characteristics of sleep patterns and disturbances in children and their parents

As shown in Table 4, children's mean bedtime was 9:51 PM [standard deviation (SD) = 45 min], mean wake-up time was 6:52 AM (SD = 24 min), mean sleep duration was 8 h and 44 min (SD = 44 min), and 41.6% of children had sleep durations less than the recommended 9 h for this age group (32). Children who needed a co-sleeper accounted for 88.1% of all children (326/370), and 59.5% of children suffered from global sleep disturbances (CSHQ total score >41). Bedtime resistance (32.2%), sleep anxiety (21.4%), and short sleep duration (20.5%) were the most prevalent sleep disturbances, followed by daytime sleepiness (9.7%), parasomnia (7.3%), sleep-disordered breathing (3.0%), sleep onset delay (0.4%), and night waking (0.2%). Furthermore, parents' mean bedtime was 11:34 PM (SD = 1 h 12 min), mean wake-up time was 6:14 AM (SD = 42 min), mean sleep duration was 6 h and 40 min (SD = 1 h 7 min), and 53.6% of parents had sleep durations shorter than the recommended 7 h (32). Their mean PSQI score was 5.5 (SD = 2.8), and 44.3% of parents had sleep disturbances (PSQI score >5.5).

**Table 4:** Characteristics of sleep patterns and disturbances in children and their parents (*n* = 370).

	Mean ± SD	Cutoff	Prevalence (%)
Sleep patterns of child h:min			
Bedtime	9:51 ± 0:45	** **	** **
Wake-up time	6:52 ± 0:24	** **	** **
Sleep duration	8:44 ± 0:44	9^a^	41. 6
Co-sleeping *n* (%)	326 (88.1%)		
Sleeping with parents	255 (78.2%)^2)^		
Sleeping only with siblings	56 (17.2%)^2)^		
CSHQ score			
Total score	43.1 ± 6.5	41^b^	59.5
Bedtime resistance	9.4 ± 2.8	10.84^c^	32.2
Sleep onset delay	1.2 ± 0.5	2.31^c^	0.4
Sleep duration	4.2 ± 1.4	5.27^c^	20.5
Sleep anxiety	5.7 ± 2.0	7.79^c^	21.4
Night waking	3.3 ± 0.8	5.29^c^	0.2
Parasomnias	8.3 ± 1.4	10.61^c^	7.3
Sleep-disordered breathing	3.2 ± 0.6	4.5°^c^	3.0
Daytime sleepiness	11.1 ± 3.2	15.24^c^	9.7
Sleep patterns of parent h, min			
Bedtime	11:34 ± 1:12		
Wake-up time	6:14 ± 0:42		
Sleep duration	6:40 ± 1:07	7^a^	53.6
PSQI score	5.5 ± 2.8	5.5	44.3

CSHQ, Children's Sleep Habits Questionnaire; PSQI, Pittsburgh Sleep Quality Index; SD, standard deviation.

Data are presented as means ± SDs or as numbers of children with percentages in parentheses, as appropriate. Sleep start and end times are given as the time on a 12-h clock, with SDs in hours and minutes.

^a^ Recommended minimum sleep duration (32).

^b^ CSHQ clinical cutoff total score (20).

^c^ Cutoff value is >2 SDs above the mean of the community sample (20, 33).

### Path diagram

As described in the statistical analysis section, after fitting multiple models in the hypothesized Figure 1, the following 11 factors were selected as variables to complete the path diagram as shown in Figure 2: CSHQ total score, sex, after-school lessons, single-mother family, children's bedtime, PSQI total score, parents' bedtime, cram school, time spent watching television, age, and living with maternal grandmother. Estimates of each path coefficient are presented in Supplementary Table S1. The model fit was moderate with an RMSEA of 0.062 and a Pr(RMSEA ≤ 0.05) of 0.179 (Shown in Supplementary Table S2.).

### Effects of factors on SDQ total score

The path analysis revealed four factors (denoted by asterisks in Figure 2) that directly affected the SDQ total score (the direct effect is shown in Table 5). In Figure 2, the influence of one factor on its neighboring factors is indicated by β. The CSHQ total score had the largest direct impact (β_1_) on the SDQ total score (direct effect = 0.23, Z score = 6.12). A higher CSHQ total score (bad sleep habits) was significantly related to a higher SDQ total score. After-school lessons had the second-largest direct impact (β_3_; direct effect = 0.23, Z score = −5.09), where attending after-school lessons was significantly related to a lower SDQ total score. Being a single-mother family had a significant effect on the SDQ total score (β4; direct effect = 3.6, Z score = 4.66), and sex had a significant effect on SDQ total score, with boys having a higher total SDQ total score than girls (β2; direct effect = −2.97, Z score = −2.26). The total effects (direct plus indirect effects) of each factor for SDQ total score are shown in Table 6. Attending after-school lessons, which had a significant direct impact (β_3_) on the SDQ total score, influenced the SDQ total score *via* the CSHQ total score (β_7_β_1_). Therefore, the total effect of attending after-school lessons on the SDQ total score (β_3 _+ β_7_β_1_) was −3.28. Similarly, being a single-mother family significantly influenced the SDQ total score *via* children's bedtime, CSHQ total score (β_9_β_5_β_1_), and PSQI total score (β_14_β_6_β_1_); the total effect of single-mother families (β_4 _+ β_9_β_5_β_1 _+ β_14_β_6_β_1_) was 3.67. Attending cram school (β_11_β_5_β_1 _= 0.033), time spent watching television (β_12_β_5_β_1 _= 0.057), and age significantly affected children's bedtime (β_13_β_5_β_1 _= 0.060) and increased the SDQ total score *via* the CSHQ total score. Furthermore, the later the parents’ bedtime, the later was the children's bedtime (β_10_β_5_β_1_) and the higher the PSQI total score (β_16_β_6_β_1_). The standardized effect shown in Table 6 demonstrates that the CSHQ total score was the most influential factor. Moreover, CSHQ had a significant impact on not only the SDQ total score but also numerous other factors. For example, after-school lessons, parental sleep, time spent watching television, and age were related to the CSHQ score and affected the SDQ score.

**Table 5:** Direct effects of factors on the SDQ total score.

Factor	Direct effect	SE	Parameters	*P*-value	95% confidence interval	Z-score
CSHQ total score	0.23	0.04	β1	<0.01	0.16	0.31	6.12
Sex	−2.97	0.49	β2	0.024	−2.06	−0.15	−2.26
After-school lessons	0.23	0.58	β3	<0.01	−4.11	−1.83	−5.09
Single-mother family	3.6	0.77	β4	<0.01	2.08	5.11	4.66

CSHQ, Children's Sleep Habits Questionnaire; PSQI, Pittsburgh Sleep Quality Index; SE, standard error.

**Table 6:** Total effects and standardized parameters of factors on the SDQ total score.

Factor	Parameters	Total effect	SE	*P*-value	Std. parameter	SE
CSHQ total score	β_1_	0.233	0.038	<0.001	0.2905	0.0453
Sex	β_2_	−1.102	0.4881	0.024	−0.1069	0.0469
After-school lessons	β_3 _+ β_7_β_1_	−3.2813	0.61	<0.001	−0.2671	0.0471
Single-mother family	β_4 _+ β_9_β_5_β_1 _+ β_14_β_6_β_1_	3.6688	0.7741	<0.001	0.2249	0.0455
Children's bedtime	β_5_β_1_	0.005847	0.002	0.003	0.0517	0.0172
PSQI total score	β_6_β_1_	0.06775	0.0312	0.03	0.0359	0.0164
Parents’ bedtime	β_10_β_5_β_1 _+ β_16_β_6_β_1_	0.0015	0.0008	0.053	0.021	0.0069
Attending cram school	β_11_β_5_β_1_	0.0332	0.0293	0.257	0.003	0.0026
Time spent watching television	β_12_β_5_β_1_	0.0567	0.0215	0.009	0.0137	0.0051
Age	β_13_β_5_β_1_	0.0595	0.0213	0.005	0.0198	0.007
Living with maternal grandmother	β_15_β_6_β_1 _+ β_8_β_1_	0.3955	0.2656	0.137	0.0234	0.0156

CSHQ, Children's Sleep Habits Questionnaire; PSQI, Pittsburgh Sleep Quality Index; SE, standard error.

Std. parameter; standardized parameter.

### Effects of the four factors (CSHQ total score, sex, after-school lessons, and single-mother family) on SDQ subscale scores

The effects of the four factors that directly affected the SDQ total score on the SDQ subscale scores are shown in Table 7. The CSHQ total score was significantly related to emotional symptoms, such as “often complains of headaches, stomachaches, or sickness” and “many worries or often seems worried,” conduct problems, such as “often loses temper” and “generally well behaved, usually does what adults request,” and hyperactivity/inattention, such as “restless, overactive, cannot stay still for long” and “constantly fidgeting or squirming.” A higher CSHQ total score was significantly related to higher SDQ subscale scores. In regard to sex differences, boys scored significantly higher than girls for conduct problems and hyperactivity and significantly lower for prosocial behavior, such as “considerate of other people’s feelings” and “shares readily with other children, for example, toys, treats, and pencils.” Attending after-school lessons was related to significantly lower subscale scores for emotional symptoms, conduct problems, hyperactivity, and peer problems. Peer problems included descriptions of “rather solitary, prefers to play alone” and “has at least one good friend.” Being a single-mother family was related to significantly higher scores for conduct problems, hyperactivity, and peer problems.

**Table 7:** Effect of the four factors on SDQ subscale scores (CSHQ total score, sex, after-school lessons, and single-mother family).

	CSHQ total score	Sex	After-school lessons	Single-mother family
Emotional symptoms	0.07**	0.25	−0.61**	0.35
Conduct problems	0.07**	−0.41*	−0.63**	1.01**
Hyperactivity	0.08**	−0.81**	−0.92**	1.30**
Peer problems	0.02	−0.30	−0.65**	0.80**
Prosocial behavior	−0.02	0.64**	0.23	−0.25

SDQ, Strengths and Difficulties Questionnaire; CSHQ, Children's Sleep Habits Questionnaire.

Data are presented as estimates. *p < 0.05, **p < 0.01.

## Discussion

This study aimed to identify the factors that have the greatest impact on children's behavioral and emotional problems, including physical discomfort, sleep status, time spent watching television, playing games, and using the internet, family structure, family employment status, and parent's sleep status, and to determine how each factor affects other factors. We used path analysis because it allows the directed dependencies to be described among a set of variables. We selected one community-based school to avoid the biases introduced by hospital-based studies. Furthermore, we obtained an extremely high response rate of 95.4%, which provided our study with high reliability. This was achieved by frequently visiting the elementary school and explaining the significance of this study to both caregivers and teachers.

In testing the hypotheses formulated in Figure 1, as shown in Figure 2, we found that age and sex as physical factors, time spent watching television and attending after-school lessons and cram school as lifestyle factors, and single-mother family and living with the maternal grandmother as nurturing environments remained as factors directly or indirectly affecting SDQ scores. The CSHQ score, parents' and children's bedtimes, and PSQI score, which represents parents' sleep quality, were also factors that were related to the SDQ score. This indicated that physical, lifestyle, and child-rearing environment factors are related to the SDQ score *via* the CSHQ score.

The path analysis revealed that the factor that most influenced the SDQ total score was the CSHQ total score, which represents sleep disturbances in children. Although several studies have reported a relationship between children's sleep quality and behavior, most have been focused on infants and children with ASD, ADHD, and sleep disorders (34–36). Moreover, most studies that used path analysis were focused on infants and children with ASD and ADHD and the association with obesity or were specific to the nurturing environment, such as poverty or the mental state of the mother (37–39). This is the first study to conduct path analysis in school-aged children, excluding those with ASD and ADHD, that included not only the childcare environment but also the lifestyle, physical illness status, and sleep status of the parents. Rather than performing a simple correlation between children's sleep disturbances and behavioral and emotional problems, we tested a structural model that included factors that affect children's behavior and emotional symptoms.

The results suggested that sleep disturbances not only directly affect children's emotions and behaviors but also mediate nurturing environments, such as family structure and parental sleep; lifestyles, such as attendance at cram school and after-school lessons, time spent watching television, and time to go to bed; and age. For example, as shown in Figure 2, although time spent watching television did not directly affect the SDQ score, longer times watching television delayed children's bedtimes and deteriorated sleep quality, which resulted in a poor SDQ score. Conversely, because television viewing time only had an indirect effect on the SDQ score, if children spent a prolonged time watching television, there was no negative impact on the SDQ score if sleep was undisturbed. In a clinical setting, healthcare providers need to consider various background factors, such as the nurturing environment, to improve children's behavioral and emotional problems. However, in practice, some of these problems cannot be solved immediately. Our study findings suggest that even if the nurturing environment and other factors cannot be addressed, initial interventions in the form of sleep hygiene guidance may positively impact children's emotions and behavior because myriad factors affect children's behavior and emotions *via* sleep. Regarding sleep and children's behavior problems, Smedje et al. found that 36% of children who report sleep problems globally are expected to have serious behavioral problems, whereas 15% of children with behavioral problems report sleep problems globally (4). Fosse et al. reported that children with higher SDQ scores are more susceptible to sleep disturbances (40). Moreover, emotional symptoms, behavioral problems, and hyperactivity have been shown to be associated with sleep disturbances when sociodemographic factors (i.e., child's sex, age, and school grade and parental education level and marital status) were controlled (40). These results are consistent with our findings that the total CSHQ score is directly related to SDQ subscale scores for emotional symptoms, behavioral problems, and hyperactivity. Longitudinal sleep disturbances reduce serotonin levels and contribute to anxiety disorder, depression, and increased suicide risk (41–43). These findings indicate that disrupted sleep affects brain function and leads to emotional and behavioral disorders in children.

Despite sleep having been shown to influence children's behavior, parents are not often aware of its importance. In our study, children's sleep duration of 8 h 45 min was considerably shorter than that of children in the United States (10 h 38 min) and China (9 h 14 min) (28). Sleep duration for the current study age group is recommended at 9–11 h, and 41.6% of children in our study had sleep durations of less than 9 h. It has been noted that Japanese people, along with Korean people, sleep less than those in other countries, and it has been reported that their average sleep duration is approximately 1 h shorter than the average sleep duration of those in other countries (44). The proportion of children who had problems with sleep duration based on the CSHQ subscale score was 20.5%, which suggests that only 20.5% of parents are aware of children's short sleeping durations, despite the high rate of children (41.6%) who do not sleep for the recommended amount of time. This indicates that half of parents with sleep-deprived children are not aware that their children are receiving inadequate sleep. In addition, we showed that parental sleep status affects children's emotions and behavior *via* sleep. Improving parents' understanding of the importance of sleep may enhance both parents' and children's sleep statuses, which, in turn, may contribute to improvements in children's emotions and behavior.

After-school lessons had the second-largest impact on the SDQ total score and directly and indirectly affected the SDQ score *via* the CSHQ total score. Participation in after-school lessons was significantly associated with lower scores on the SDQ subscales for emotional symptoms, conduct problems, hyperactivity, and peer problems. In Japan, more than 70% of children in elementary school attend at least one after-school lesson, which is consistent with our result of 74.8% (45). These lessons can be divided into two major activities: physical (e.g., swimming, baseball, and soccer) and cultural activities (e.g., piano and Japanese calligraphy). Most Japanese parents expect their children to not only master skills and develop physical strength but also gain self-confidence and learn cooperation, good manners, and concentration through enjoying sports or cultural activities. Hansen et al. reported that participating in cultural activities is positively associated with health, life satisfaction, and self-esteem in adolescents, which may be explained by social relationships (46). Furthermore, fewer internalizing problems and better prosocial behavior are exhibited by children who participate in sports (47). Compared with children who stay home after school, those who spend time with peers after school have been shown to have a lower probability of scoring high on the SDQ (26) and higher self-esteem (48). In Japan, parents experience time and financial burdens for their children's participation in after-school lessons (49). Herrmann et al. reported that economic conditions affect the SDQ score (10); however, we did not collect data on the economic status of the families. Moreover, parents' mental symptoms have also been reported to affect the SDQ score; thus, parents' time allowance may also affect the SDQ score (9). In Japan, child custody is transferred to one parent following a divorce and, in most cases, the mother retains custody. According to a survey by the Ministry of Health, Labour and Welfare, 24.5% of cases receive regular payments for child support; however, more than half (56.5%) of cases never receive child support from the father, which has resulted in poverty among single-mother families becoming a social problem (50). Therefore, single-mother families are unlikely to have sufficient time or financial resources, which may have contributed to the poor SDQ scores in these children. Sex differences in the total SDQ score and SDQ subscale scores have been documented previously; the original study conducted in the United Kingdom and other subsequent studies have shown that boys exhibit more difficulties than girls, except for emotional symptoms (12, 18, 19). Furthermore, Rescorla et al. reported that girls score significantly higher than boys for internalizing problems, whereas boys score significantly higher than girls for externalizing problems (25). When assessing a child's behavior, healthcare providers and supporters should consider factors that directly or indirectly influence the child's behavior, such as after-school activities and family structure.

This study had several limitations. First, the study results were based on subjective data owing to the use of parent-reported instruments. Second, the original CSHQ has not been validated in children aged 11 years and older. Third, we did not examine family income to assess the economic status of the families.

## Conclusion

Our study indicated that children's sleep disturbances had the largest impact on the SDQ total score, and participation in after-school lessons had the second-largest impact on the SDQ total score. Most factors affected children's behavior through their sleep quality, which suggests that children's behavioral problems can be improved by not only interventions that directly target behavior but also those that focus on sleep quality, such as sleep hygiene instructions. Longitudinal studies are needed to identify other factors that affect the SDQ score, such as family economic status, parental mental state, and social media circumstances. Our findings are clinically significant in terms of teaching children with behavioral and emotional problems to “sleep well at night” over instructing them to “work hard and do it right.”

## Data Availability

The raw data supporting the conclusions of this article will be made available by the authors, without undue reservation.
