# Supplementary material for: Sleep disturbance has the largest impact on children's behavior and emotions
Source: Front Pediatr. 2022 Nov 28;10:1034057. doi: 10.3389/fped.2022.1034057 (PMC9744255; doi:10.3389/fped.2022.1034057)
Supplement: Supplementary file 1 [file Datasheet1.docx]

Supplementary Material

**Supplementary Table1. Estimates of each path coefficient**

| **Structural model** |  | **Coef.** | **SE** | **z** | **P > z** | **95% CI** | |
| --- | --- | --- | --- | --- | --- | --- | --- |
| **SDQ total score** | Y1 |  |  |  |  |  |  |
| **CSHQ total score** | 1 | 0.23 | 0.04 | 6.12 | 0.000 | 0.16 | 0.31 |
| **Sex** | β_2_ | −1.1 | 0.49 | −2.26 | 0.024 | −2.06 | −0.15 |
| **After-school lessons** | β_3_ | −2.97 | 0.58 | −5.09 | 0.000 | −4.11 | −1.83 |
| **Single-mother family** | β_4_ | 3.6 | 0.77 | 4.66 | 0.000 | 2.08 | 5.11 |
| cons |  | 2.43 | 1.86 | 1.31 | 0.190 | −1.21 | 6.07 |
| **CSHQ total score** | Y2 |  |  |  |  |  |  |
| **Children’s bedtime** | β_5_ | 0.03 | 0.01 | 3.39 | 0.001 | 0.01 | 0.04 |
| **PSQI total score** | β_6_ | 0.29 | 0.12 | 2.33 | 0.020 | 0.05 | 0.53 |
| **After-school lessons** | β_7_ | −1.34 | 0.8 | −1.68 | 0.093 | −2.91 | 0.22 |
| **Living with maternal grandmother** | β_8_ | 1.41 | 1.11 | 1.28 | 0.202 | −0.76 | 3.58 |
| cons |  | 42.69 | 1.02 | 41.97 | 0.000 | 40.7 | 44.69 |
| **Children’s bedtime** | Y3 |  |  |  |  |  |  |
| **Single-mother family** | β_9_ | 1.5 | 6.61 | 0.23 | 0.821 | −11.47 | 14.46 |
| **Parents’ bedtime** | β_10_ | 0.12 | 0.03 | 3.91 | 0.000 | 0.06 | 0.17 |
| **Cram school** | β_11_ | 5.68 | 4.62 | 1.23 | 0.220 | −3.39 | 14.74 |
| **Time spent watching television** | β_12_ | 9.69 | 1.69 | 5.73 | 0.000 | 6.37 | 13 |
| **Age** | β_13_ | 10.17 | 1.23 | 8.24 | 0.000 | 7.75 | 12.59 |
| cons |  | −123.62 | 11.51 | −10.74 | 0.000 | −146.17 | −101.06 |
| **PSQI total score** | Y4 |  |  |  |  |  |  |
| **Single-mother family** | β_14_ | 0.95 | 0.46 | 2.08 | 0.037 | 0.06 | 1.85 |
| **Living with maternal grandmother** | β_15_ | 0.98 | 0.47 | 2.07 | 0.039 | 0.05 | 1.9 |
| **Parents’ bedtime** | β_16_ | 0.01 | 0 | 6.54 | 0.000 | 0.01 | 0.02 |
| cons |  | 5.24 | 0.15 | 35.44 | 0.000 | 4.95 | 5.53 |

SDQ, Strengths and Difficulties Questionnaire; CSHQ, Children’s Sleep Habits Questionnaire; PSQI, Pittsburgh Sleep Quality Index; SE, standard error; Coef., coefficient; CI, confidence interval; cons, constant

**Supplementary Table2. Goodness of Fit Statistics.**

|  | **Fit statistic** | **Value** | **Description** |
| --- | --- | --- | --- |
| **Likelihood ratio** | χ^2^ (df = 22), *p*-value | 50.874, 0 | Model vs. saturated |
|  | χ^2^ (df = 38), *p*-value | 330.59, 0 | Baseline vs. saturated |
| **Population error** | RMSEA | 0.062 |  |
|  | 90% CI | (0.039, 0.084) |  |
|  | pclose | 0.179 | Probability RMSEA ≤ 0.05 |
| **Information criteria** | AIC | 17485.195 |  |
|  | BIC | 17577.579 |  |
| **Baseline comparison** | CFI | 0.901 |  |
|  | TLI | 0.83 |  |
| **Size of residuals** | SRMR | 0.042 |  |
|  | CD | 0.476 |  |

df, degree of freedom; RMSEA, Root mean squared error of approximation; CI, Confidence Interval; AIC, Akaike information criterion; BIC, Bayesian information criterion; CFI, Comparative fit index; TLI, Tucker-Lewis index; SRMR, Standardized root mean squared residual; CD, Coefficient of determination
